# Predictive values of Mallampati score, tonsillar size, and BMI z-score in the presence and severity of obstructive sleep apnea in pediatric population

**DOI:** 10.55730/1300-0144.5791

**Published:** 2023-12-08

**Authors:** Ayşe KAÇAR BAYRAM, Ahu PAKETÇİ, Melek Nur ŞAHBAZ, Sevde DAŞDELEN, Serkan Bilge KOCA

**Affiliations:** 1 Division of Pediatric Neurology, Department of Pediatrics, Kayseri City Training and Research Hospital, University of Health Sciences, Kayseri, Turkiye; 2 Division of Pediatric Neurology, Department of Pediatrics, Etlik City Training and Research Hospital, Ankara, Turkiye; 3 Division of Pediatric Endocrinology, Department of Pediatrics, Kayseri City Training and Research Hospital, University of Health Sciences, Kayseri, Turkiye

**Keywords:** Children, obstructive sleep apnea, Mallampati score, BMI z-score, tonsillar size

## Abstract

**Background/aim:**

Obstructive sleep apnea (OSA) is a common sleep-related breathing disorder in children. Determination of risk factors for the development of OSA is essential for early diagnosis and treatment of the disease and decreases the risk of negative consequences. This study aimed to investigate the predictive values of Mallampati score, tonsillar size, and BMI z-score in the presence and severity of OSA in children.

**Materials and methods:**

This prospective cross-sectional study included 114 children with OSA symptoms. All children were assessed by BMI z-score, Mallampati score, and tonsillar size and underwent overnight polysomnography. They were consecutively selected and assigned to 4 groups as follows: Group 1 included normal-weight with a low Mallampati score; Group 2 involved normal-weight with a high Mallampati score; Group 3 included obese with a low Mallampati score; and Group 4 involved obese with a high Mallampati score.

**Results:**

Of the 114 included children, 58 were female and 56 were male, with a mean age of 13.1 ± 2.9 years. OSA frequency and apnea-hypopnea index were significantly higher in group 4 compared with other groups (p = 0.003 and p < 0.0001, respectively), whereas average and minimum spO_2_ were significantly lower (for both, p = 0.001). Mallampati score and BMI z-score were found to be significant for predicting OSA (odds ratio = 4.147, 95% CI: 1.440–11.944; p = 0.008 and odds ratio = 1.760, 95% CI: 1.039–2.980; p = 0.035, respectively). Among OSA patients, the Mallampati score, tonsillar size, and BMI z-score were found to be significant for predicting OSA severity (odds ratio = 4.520, 95% CI: 1.332–15.335, p = 0.015, odds ratio = 9.177, 95% CI: 2.513–33.514, p = 0.001, and odds ratio = 2.820, 95% CI: 1.444–5.508; p = 0.002, respectively).

**Conclusion:**

The coexistence of the Mallampati score and BMI z-score significantly increases the presence of OSA in children. Mallampati score, tonsillar size, and BMI z-score are promising parameters for predicting OSA severity.

## 1. Introduction

Obstructive sleep apnea (OSA) is a common pediatric breathing disorder in sleep, characterized by the upper airway’s episodic collapse, snoring, intermittent nocturnal gas exchange abnormalities, and sleep disruption. Although OSA affects around 1%–4% of healthy children [1], its prevalence in obese children is up to 60% [2]. The potential consequences of untreated pediatric OSA are neurobehavioral disorders, learning deficits, pulmonary and/or systemic hypertension, and endocrinological disturbances [3]. The questionnaires, history, and physical examination findings have been used to predict pediatric OSA but mainly present low sensitivity and specificity [4]. The gold standard in the diagnosis and severity of OSA is polysomnography (PSG) [5].

The prevalence and severity of pediatric obesity have increased dramatically over the last few decades [6]. An increase in the pharyngeal soft adipose tissue is the leading cause of respiratory disturbances during sleep in obese children. Increased adipose tissue decreases the lumen’s caliber, causing the tendency of the structures’ obstruction. In addition, the adipose tissues of the thoracic and abdominal walls significantly decrease lung and chest wall compliance [2, 7]. Mallampati classification has been used to assess difficult intubation over three decades, determining the tongue size relative to the oropharyngeal cavity [8]. A high Mallampati score could be related to a strong possibility of sleep-related breathing disorder because the upper airway patency is smaller [9].

Several studies have different results regarding the utility of obesity and the Mallampati score in the prediction and severity of OSA. However, no studies in the pediatric population have compared patients who have coexisting these two clinical conditions. Herein, we present predictive values of the Mallampati score, tonsillar size, and BMI z-score for the presence and severity of OSA in different groups of the pediatric population.

## 2. Materials and methods

### 2.1. Study design and participants

This prospective cross-sectional study was carried out in the Pediatric Neurology and Sleep Medicine Department at Kayseri City Education and Research Hospital between February 2019 and January 2022, including 114 children with OSA symptoms such as unrefreshing sleep, daytime sleepiness, fatigue or insomnia, distractibility, interrupted sleep, snoring, or witnessed apneas. The local ethics committee approved this study project (#566/2021), and it stuck with the criteria of the Declaration of Helsinki’s tenants. All children and their parents/guardians were informed before their enrolment in the study, and written informed consent was gained. Fifty-six obese children with OSA symptoms were consecutively involved from the Pediatric Endocrinology Department of the same hospital or the patients referred to our Center who had a body mass index (BMI) ≥95 percentile according to the Centers for Disease Control and Prevention (CDC) 2000 growth charts and curves except for children with endocrinological problems, genetic abnormalities, medical agents, or organ dysfunction-related obesity. Fifty-eight normal-weight children with a BMI above the 5^th^ and below the 85^th^ percentile were consecutively involved among the patients referred to our Center for Assessment and Management of OSA between February 2019 and January 2022. The exclusion criteria for both groups were chronic lung disorders, neuromuscular disease, craniofacial abnormalities, down syndrome, adenotonsillar surgery, and laryngeal and/or tracheal malformations. All participants underwent a comprehensive physical examination, including weight and height measurements, tonsillar size, Mallampati score, and full-night polysomnography, and were classified into four groups based on the Mallampati score and/or obesity presence.

### 2.2. Physical examination

Clinical characteristics, including age, sex, height, weight, Mallampati score, and tonsillar size, were obtained from all participants. BMI (kg/m^2^) and BMI z-score were calculated using height and weight. BMI standard deviation score (SDS) and percentiles were calculated based on the CDC 2000 growth charts and curves [10]. BMI > 95^th^ percentile was accepted as pediatric obesity, and normal weight was determined as a BMI between the 5^th^ and 85^th^ percentile, according to the CDC [11]. The lambda (L)-mu (M)-sigma (S) method was used to calculate BMI z-scores based on BMI z-score = (BMI/M)L-1/LS equation [12]. In this equation, L shows the Box-Cox power transform required to eliminate the skewed distribution, M represents the median BMI by age, and S refers to the coefficient of variation.

Mallampati score was evaluated in the upright sitting position according to the Modified Mallampati classification [13]. It was made based on the visibility of anatomical structures of the oropharynx as follows: grade I: tonsillar pillars, fauces, soft palate, and uvula are visible; grade II: anterior tonsillar pillar, fauces, soft palate, and a part of the uvula are seen; grade III: uvula base, soft and hard palate are seen; grade IV: the hard palate is only visible [13]. Grades I and II were considered low Mallampati scores, and grades III and IV were accepted as high. Brodsky tonsil grading was performed to determine tonsillar size [14]. Each child was requested to open the mouth as big as possible and protrude the tongue as far as possible. If the individual had a high Mallampati score, the tongue was depressed by a tongue depressor to allow for the examination of the tonsils. This scale grades tonsil size into 4 degrees as follows: degree 0: tonsils are seen in the fossa; degree 1: tonsils cover the oropharynx less than 25%; degree 2: tonsils cover 25%–50% of the oropharynx; degree 3: tonsils cover 50%–75% of the oropharynx; degree 4: tonsils cover the oropharynx more than 75%. Two independent masked and experienced pediatricians assessed the Mallampati score and tonsil size. In case of disagreement between the two examiners, the evaluation was made by a third pediatrician to decrease potential bias.

### 2.3. Classification of the study population

All participants were assigned to 4 groups: Group 1 included normal-weight children with a low Mallampati score; Group 2 involved normal-weight children with a high Mallampati score; Group 3 had obese children with a low Mallampati score; Group 4 involved obese children with a high Mallampati score.

### 2.4. Overnight polysomnography

Overnight polysomnography was performed using Alice 6 LDxS™ computerized system (Philips Respironics Inc., PA, USA) at our Hospital’s Pediatric Sleep Unit, following the American Academy of Sleep Medicine guidelines [15]. All participants were investigated in a quiet, darkened room with about 24 °C environment temperature, accompanied by a parent or guardian for up to 12 h. The monitoring consisted of an electroencephalogram using the electrode placement according to the International 10–20 System, an electrocardiogram, peripheral oxygen saturation with a pulse oximeter, submental and anterior tibial electromyogram, a bilateral electrooculogram, airflow monitoring by the oronasal thermistor and a nasal pressure transducer, vibration detector, monitoring upper respiratory tract sound by sonography, abdominal and thorax plethysmography, and positions’ video surveillance and movements of the body. Analysis was obtained in 30-s epochs, and all of them were evaluated. If children had an unsuccessful sleep examination (sleep time <4.00 h, sleep efficiency <65%, and/or signal quality <90%), they were reassessed with PSG. The apnea index (AI) was determined as the sum of apneas divided by the whole sleep time, while central apneas were excluded. The apnea-hypopnea index (AHI) was defined as the total number of apneas plus hypopneas per recording sleep hour, while central apneas were excluded. Hypopnea was represented by a decrease of ≥50% of the airflow signal’s amplitude, which was quantified when longer than two baseline breaths and was related to oxygen desaturation of at least 4% and/or arousals. The arousal index represented the total number of arousals per hour of sleep. Children with AHI ≥1 were considered to have OSA. OSA severity was graded based on current clinically accepted criteria using an AHI of ≥1 to <5 for mild OSA, ≥5 to <10 for moderate OSA, and ≥10 for severe OSA [16].

### 2.5. Statistical analysis

The SPSS Statistics software version 26.0 for Mac OS (SPSS Inc, Chicago, IL, USA) analyzed the data. Categorical and continuous variables were presented as frequency distributions/percentages and the mean ± standard deviation, respectively. The categorical variables were evaluated using Pearson’s chi-square and Fisher’s exact test. Pearson’s Chi-square post hoc analysis was assessed based on the adjusted residuals with the Bonferroni correction. The Kolmogorov-Smirnov test evaluated the normal distribution of the variables. Levene’s test assessed the homogeneity of variances. The one-way variance analysis was performed to compare continuous variables between the four groups. When a significant result was obtained, the Tukey test was performed for post hoc comparisons. Kruskal Wallis-H test with pairwise comparisons by the Bonferroni correction evaluated the nonnormally distributed data. A binary logistic regression analysis was used to determine the clinical predictors of OSA and OSA severity. A p-value less than 0.05 was accepted as statistically significant.

## 3. Results

**Figure 1:**
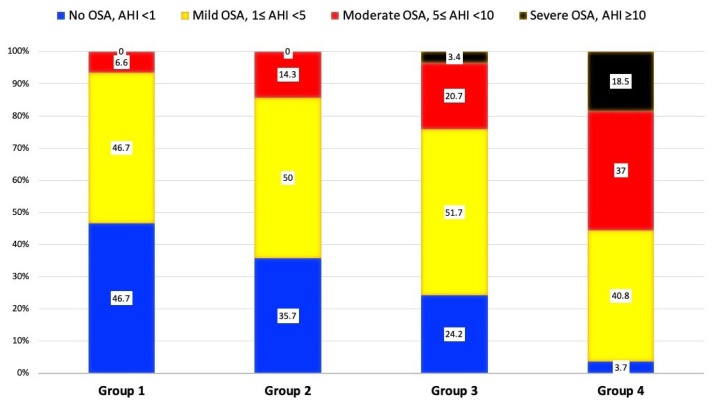
A stacked bar graph shows the distribution of the severity of obstructive sleep apnea among the groups.

Of the 114 children included in the study, 58 (50.9%) were girls and 56 (49.1%) were boys, with a mean age of 13.1 ± 2.9 years. All children were divided into four groups as follows: group 1 (n = 30, 26.3%), group 2 (n = 28, 24.6%), group 3 (n = 29, 24.4%), and group 4 (n = 27, 23.7%). No significant difference was observed between the groups concerning age (p = 0.841), sex (p = 0.841), and tonsillar size (p = 0.912). Groups 1 and 3, and groups 2 and 4 had similar anatomy estimated by the modified Mallampati score (p = 0.785 for groups 1 and 3, and p = 0.943 for groups 2 and 4). Similarly, there was no significant difference in the BMI z-scores between groups 1 and 2, and between groups 3 and 4 (p = 0.744 for groups 1 and 2, and p = 0.073 for groups 3 and 4). In addition, there were no statistically significant differences in the sleep parameters, including total sleep time, sleep efficiency, sleep latency, REM sleep, NREM-1 sleep, NREM-2 sleep, NREM-3 sleep, and arousal index among the four groups (for all, p > 0.05). There was no statistically significant difference in the AHI among groups 1–3, but group 4 showed a significantly higher AHI than in other groups (p < 0.0001) (Figure 1). Also, the average spO_2_ and minimum spO_2_ did not change significantly among groups 1–3, but they were significantly lower in group 4 compared with group 1 (for both, p = 0.001). The study subjects’ clinical characteristics and AHI results are shown in Table 1.

**Figure 2:**
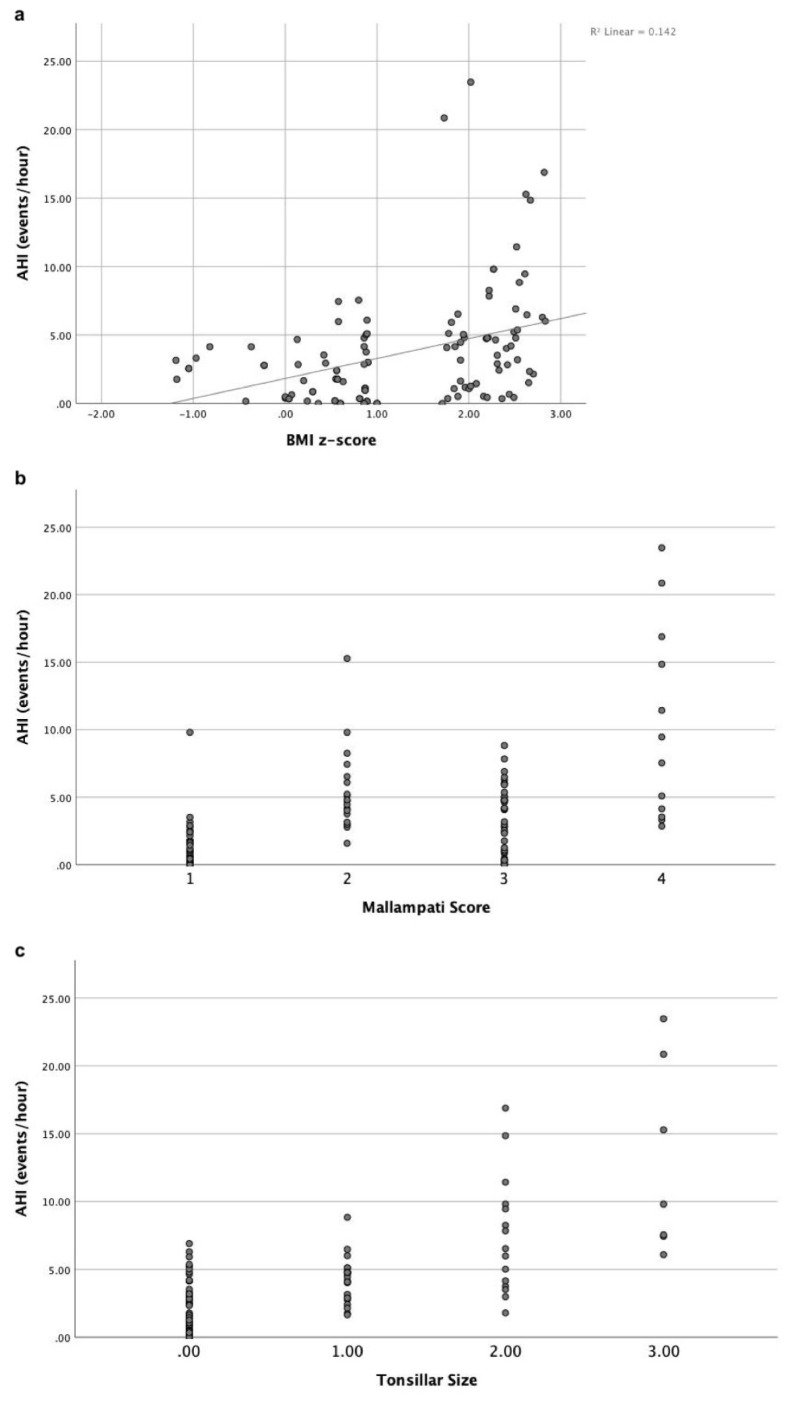
Composite scatter plots are represented apnea-hypopnea index by body mass index z-score (a), the modified Mallampati score (b), and tonsillar size by the Brodsky grading scale (c).

Eighty-two children (71.9%) were diagnosed with OSA, and 32 children (28.1%) did not have OSA. The OSA was observed in 16 patients (53.3%) of Group 1, 18 patients (64.3%) of Group 2, 22 patients (75.8%) of Group 3, and 26 patients (96.3%) of Group 4. OSA frequency was statistically significantly higher in group 4 than in other groups (p = 0.003) (Figure 1). The study population’s demographic, anthropometric, and polysomnographic findings are summarized in Table 2 (Figures 2a–2c). A binary logistic regression evaluated OSA prediction with Mallampati score, tonsil size, and BMI z-score as independent variables. Mallampati score and BMI z-score predicted OSA significantly. For every point rise in the Mallampati score, the odds ratio of OSA existence rose by 4.147-fold (95% confidence interval: 1.440–11.944; p = 0.008). For every point increase in the BMI z-score, the odds ratio of OSA existence rose by 1.760-fold (95% confidence interval: 1.039–2.980; p = 0.035).

When only patients diagnosed with OSA were evaluated, AHI and arousal index were significantly higher in group 4 compared with group 1 (3.0 ± 1.7 events/h vs. 7.5 ± 5.7 events/h, p = 0.0002 for AHI and 8.8 ± 3.4 events/h vs. 14.9 ± 6.8 events/h, p = 0.002 for arousal index). Among OSA patients, moderate and severe OSA (AHI ≥5) was also predicted by logistic regression analysis using the Mallampati score, tonsillar size, and BMI z-score as independent variables. All independent variables in the equation were found to be significant for predicting OSA severity. For every point rise in the Mallampati score, the odds ratio of OSA severity rose by 4.520-fold (95% confidence interval: 1.332–15.335, p = 0.015). For every point increase in the tonsillar size, the odds ratio of OSA severity rose by 9.177-fold (95% confidence interval: 2.513–33.514, p = 0.001). For every point increase in the BMI z-score, the odds ratio of OSA severity rose by 2.820-fold (95% confidence interval: 1.444–5.508; p = 0.002). The results of a binary logistic regression model are summarized in Table 3.

## 4. Discussion

Good clinical diagnostic indicators are essential to help examine and screen children with a risk of developing OSA to use early therapeutic interventions, thereby decreasing the risk of negative consequences. Because increased upper airway resistance is critical in developing OSA while asleep, investigating anatomic measures of the airway lumen, soft tissue, and skeleton is critical. This study focused on the utility of the Mallampati score, tonsillar size, and BMI z-score for predicting the presence and severity of OSA in the pediatric population and confirmed that several factors could be used as predictors for OSA presence and severity. The study results showed that the Mallampati score and BMI z-score were independent predictor factors for OSA presence in children and were also predictor factors for OSA severity (Table 3). AHI was significantly higher in patients who have high-grade Mallampati and obesity coexistence (group 4) compared with other groups (Table 2). Also, the tonsillar size strongly predicted OSA severity in patients with OSA (Table 3).

It is generally accepted that increased BMI, large tonsil size, and high Mallampati score are associated with an increased risk of OSA. However, the evidence about the relationship of these factors with the severity of OSA is controversial. Two recent studies showed the association between a modified Mallampati score and tonsil size with AHI in children [9, 17], which concurs with our results. Villa et al. [18] reported that a combined evaluation of clinical examination, OSA symptoms, and patient history has a strong predictive value, at least in identifying children at low risk of developing a severe OSA form. Zreaqat et al. [19] showed a correlation between the Mallampati score and OSA severity, but they did not detect with BMI or tonsillar size. The authors conclude that only the Mallampati scale had a significant relationship with OSA severity. On the other hand, Brietzke et al. [20] found that clinical history and physical examination of patients are unreliable for OSA diagnosis compared with overnight PSG after reviewing the literature on the pediatric population.

Similar to several previous studies, we found no relation between the patient’s age and OSA severity [9, 17–19]. Also, we did not detect a significant difference in OSA presence between boys and girls, which agrees with most previous studies [9, 17, 18]. However, Zreaqat et al. [19] reported that the male sex is significantly higher in OSA patients.

There is a relationship between increased obesity and OSA in the pediatric population [21]. The fat tissue in the tongue and neck area may decrease the usual tone of respiratory muscles developed during respiration, alter the functional residual capacity, and lead to mechanical airflow restriction. This study showed that the BMI z-score predicted OSA presence and severity in children. Since BMI may not precisely reflect adiposity and obesity in children, we chose to examine associations with BMI z-score to allow for a more accurate discernment of the influence of obesity in this study. In accordance with our results, a recent study reported that children with obesity had a significantly higher prevalence of OSA, and an increased BMI z-score was associated with increased AHI in the generalized linear regression [21]. On the other hand, Sardón et al. [22] showed no association between BMI z-score and OSA in children. These different results may be associated with race and ethnicity because a multicenter study investigated the associations between the AHI and these risk factors across ethnicities and sexes within sleep clinics. The authors found ethnic and sex variations in associations between obesity and OSA. For example, the authors reported that South American patients show greater AHI increases compared to African Americans with similar BMI increases [23].

The role of arousal in OSA pathogenesis and the pathophysiology of its complications is not fully clarified. In this study, the arousal index was significantly higher in group 4 than in group 1 (8.8 ± 3.4 events/h vs. 14.9 ± 6.8 events/h, p = 0.002) among patients diagnosed with OSA. These results are compatible with the results of Huang et al. [24]. Increased arousal frequency in patients with OSA suggests the severity of the disease.

One shortcoming of this study is that even though OSA usually occurs during sleep, tonsil size and Mallampati score were evaluated in the upright sitting position. Camacho et al. [25] showed that the upper airways’ minimum cross-sectional area decreased significantly when the patient was scanned with cone-beam computed tomography in a supine position than an upright position because of a backward displacement of the tongue base and epiglottis in the supine position.

In conclusion, the results of this study showed that the Mallampati score and BMI z-score were essential predictors for OSA presence in children and were also predictor factors for OSA severity. Also, the tonsillar size was a strong predictor of OSA severity. AHI was significantly higher in patients who have high-grade Mallampati and obesity coexistence. A comprehensive sleep history, BMI z-score, Mallampati score, and tonsillar size are promising parameters for evaluating children suspected of having OSA.

## Figures and Tables

**Table 1:** Clinical characteristics and apnea-hypopnea index findings of the study population

Clinical characteristics	Score	Group 1 (n = 30, 26.3%)	Group 2 (n = 28, 24.6%)	Group 3 (n = 29, 24.4%)	Group 4 (n = 27, 23.7%)
Body Mass Index percentiles	Mean ± SD	63.1 ± 21.4	64.3 ± 21.2	98.2 ± 1.2	98.3 ± 1.4
Tonsillar size (Brodsky Classification)	Degree 0, n (%)	18 (60.0%)	21 (75.0%)	16 (55.2%)	14 (51.9%)
	Degree 1, n (%)	6 (20.0%)	4 (14.3%)	7 (24.1%)	6 (22.2%)
	Degree 2, n (%)	4 (13.3%)	2 (7.1%)	4 (13.8%)	5 (18.5%)
	Degree 3, n (%)	2 (6.7%)	1 (3.6%)	2 (6.9%)	2 (7.4%)
Modified Mallampati score	Grade 1, n (%)	21 (70.0%)	0 (0.0%)	19 (65.5%)	0 (0.0%)
	Grade 2, n (%)	9 (30.0%)	0 (0.0%)	10 (34.5%)	0 (0.0%)
	Grade 3, n (%)	0 (0.0%)	22 (78.6%)	0 (0.0%)	21 (77.8%)
	Grade 4, n (%)	0 (0.0%)	6 (21.4%)	0 (0.0%)	6 (22.2%)
Apnea-hypopnea index (AHI)	AHI <1, n (%)	14 (46.7%)	10 (35.7%)	7 (24.1%)	1 (3.7%)
	AHI 1–4.9, n (%)	14 (46.7%)	14 (50.0%)	15 (51.7%)	11 (40.8%)
	AHI 5–9.9, n (%)	2 (6.6%)	4 (14.3%)	6 (20.7%)	10 (37.0%)
	AHI >10, n (%)	0 (0.0%)	0 (0.0%)	1 (3.5%)	5 (18.5%)

**Table 2:** Demographic, anthropometric, and polysomnographic data of the 114 children with OSA symptoms

Variables	Group 1 (n = 30, mean ± SD)	Group 2 (n = 28, mean ± SD)	Group 3 (n = 29, mean ± SD)	Group 4 (n = 27, mean ± SD)	p-value*
Age, years	12.7 ± 3.1	12.9 ± 2.8	13.1 ± 3.2	13.5 ± 2.7	0.841α
Sex (M/F)	14 (46.7%)/16 (53.3%)	14 (50.0%)/14 (50.0%)	12 (41.4%)/17 (58.6%)	16 (40.7%)/11(59.3%)	0.595β
Height, cm	154.0 ± 15.4	155.6 ± 14.3	158.0 ± 15.0	160.6 ± 12.8	0.170α
Weight, kg	48.3 ± 13.0	49.4 ± 11.9	79.2 ± 23.2	88.4 ± 22.4	**<0.0001**α **(<0.0001**, a **<0.0001**^b^, **<0.0001**, c **<0.0001**^d^**)**
BMI, kg/m^2^	19.7 ± 2.3	19.9 ± 2.2	30.9 ± 5.0	33.8 ± 5.5	**<0.0001**α **(<0.0001**, a **<0.0001**^b^, **<0.0001**, c **<0.0001**^d^**)**
BMI Z-score	0.35 ± 0.55	0.39 ± 0.57	2.18 ± 0.28	2.32 ± 0.35	**<0.0001**α **(<0.0001**, a **<0.0001**^b^, **<0.0001**, c **<0.0001**^d^**)**
REM sleep, %	11.4 ± 4.5	11.1 ± 4.3	10.1 ± 4.2	10.8 ± 4.2	0.541γ
NREM-1 sleep, %	4.6 ± 2.1	5.0 ± 2.5	6.5 ± 3.4	5.6 ± 2.5	0.733γ
NREM-2 sleep, %	40.6 ± 9.2	42.2 ± 9.3	45.3 ± 8.4	41.6 ± 8.0	0.195γ
NREM-3 sleep, %	26.6 ± 8.9	25.3 ± 9.0	21.1 ± 6.0	21.1 ± 6.1	0.139γ
Total sleep time (min)	376.1 ± 40.2	379.6 ± 45.9	389.9 ± 51.8	381.4 ± 58.7	0.746γ
Sleep efficiency, %	83.2 ± 8.9	83.5 ± 9.8	81.2 ± 7.7	79.9 ± 8.6	0.376γ
Sleep latency, min	25.5 ± 18.1	25.7 ± 20.6	36.5 ± 16.9	35.1 ± 22.7	0.056γ
Arousal index, events/h	9.9 ± 7.6	10.7 ± 8.0	12.1 ± 6.4	14.4 ± 7.1	0.105γ
AHI, events/h	1.73 ± 1.85	2.52 ± 2.09	3.61 ± 3.55	7.23 ± 5.73	**<0.0001**α **(<0.0001**, c **<0.0001**, d **0.002**,^e^**)**
Mean SpO2, %	94.9 ± 1.3	94.6 ± 1.3	93.7 ± 1.8	92.9 ± 2.0	**<0.0002**α **(0.001**^c^**)**
Minimum spO2, %)	89.7 ± 3.5	88.8 ± 3.7	87.9 ± 3.5	85.8 ± 3.0	**<0.001**α **(0.001**^c^**)**
Presence of OSA, n (%)	16 (53.3%)	18 (64.3%)	22 (75.8%)	26 (96.3%)	**0.003** β

AHI: Apnea-hypopnea index, NREM: non-rapid eye movement sleep, O REM: rapid eye movement sleep, OSA: obstructive sleep apnea

* p-value < 0.05 was considered as statistical significance. The statistically significant p-values of the pairwise comparisons are shown in brackets. Statistically significant p*-*values are shown in bold and italic font.

α Kruskal Wallis-H test with pairwise comparisons by the Bonferroni correction.

β Pearson’s Chi-square with post hoc analysis based on the adjusted residuals with the Bonferroni correction.

γ One-way analysis of variance with Tukey’s post hoc comparisons.

a Comparison between group 1 and group 3

b Comparison between group 2 and group 3

c Comparison between group 1 and group 4

d Comparison between group 2 and group 4

e Comparison between group 3 and group 4

**Table 3:** Clinical predictors of the presence and severity of obstructive sleep apnea.

Parameters	Predictor	Odds Ratio	95% Confidence Interval	p-values
OSA presence	Age	0.953	0.765–1.187	0.667
	Sex	1.589	0.482–5.237	0.446
	BMI z-score	1.760	1.039–2.980	0.035
	Mallampati score	4.147	1.440–11.944	0.008
OSA severity	Age	0.997	0.756–1.313	0.981
	Gender	2.150	0.414–11.154	0.362
	BMI z-score	2.820	1.444–5.508	0.002
	Mallampati score	4.520	1.332–15.335	0.015
	Tonsillar size	9.177	2.513–33.514	0.001

**BMI:** body mass index, **OSA**: obstructive sleep apnea
